# Essentials of Uro-Radiology

**Published:** 1991-09

**Authors:** J. E. Kabala


### ESSENTIALS OF URORADIOLOGY
**E. Stephen Amis Jr. and Jeffrey H. Newhouse**
pp xiii + 427, 1991 (Little, Brown and Co), £53.00

The authors' goal, clearly stated in their preface, is to provide an up to date concise textbook of uroradiology, to occupy the niche between multivolume treatises and passages in general texts, aimed at radiology and urology residents.

This book certainly is pleasingly modern and is prolifically illustrated with generally good quality images of virtually all modalities including MRI and transrectal ultrasound scans. The only weaknesses here are the poor quality of some of the trans-abdominal ultrasound images and the deliberately brief coverage of nuclear medicine studies which the authors consider a specialist field beyond the scope of this textbook; an opinion that would find both supporters and opponents in this country.

The text is logically written, easy to read and contains the occasionally dry piece of moral guidance ('it is also incumbent on the radiologist to be aware of the disputed issues since, in medicolegal practice, rigorous science and "expert" legal testimony may not coincide').

The first few chapters deal with the background to uroradiology and cover history (very briefly), anatomy (a useful terse refresher with good sections on retroperitoneal anatomy and the zones of the prostate), techniques (good brief review of MRI; duplex ultrasound confined to one sentence) and contrast agents (nice concise discussion).

The subsequent chapters dealing with disease are organised largely on a regional basis (the retroperitoneum, the adrenal, the kidney:cystic disease etc.). Conditions are dealt with logically (with discussions of pathology, clinical presentation and imaging techniques) and reasonably comprehensively, given the desire to keep the book as short as possible without avoiding major omissions. The bibliography is generally good (although predictably very American) and up to date. The authors have deliberately, and very reasonably, emphasised diagnostic uroradiology and avoided extensive consideration of interventional techniques. I found the index adequate and easy to use but occasionally idiosyncratic (chyluria is included but haematuria is not).

I believe the authors have achieved their target as stated at the outset. This is an elegant useful book that is informative and a pleasure to read. I would recommend it to any radiologist developing an interest in uroradiology or indeed any general radiologist who finds that a reasonable part of his workload involves investigation of the urinary tract. It is likely to be useful to urologists, especially during their training. Pre-fellowship radiologists would find it excellent for the fellowship examination; if nothing else they would be reminded, in the words of the authors, that 'good radiological practice requires the performance of necessary examinations just as it requires the avoidance of needless ones'.

J. E. Kabala

